# Plasma miRNAs as Biomarkers to Identify Patients with Castration-Resistant Metastatic Prostate Cancer

**DOI:** 10.3390/ijms14047757

**Published:** 2013-04-10

**Authors:** Akira Watahiki, Robyn J. Macfarlane, Martin E. Gleave, Francesco Crea, Yuzhuo Wang, Cheryl D. Helgason, Kim N. Chi

**Affiliations:** 1 The Vancouver Prostate Centre, Vancouver General Hospital, Vancouver, BC V6H 3Z6, Canada; E-Mails: awatahiki@bccrc.ca (A.W.); m.gleave@ubc.ca (M.E.G.); kchi@bccancer.bc.ca (K.N.C.); 2 Departments of Experimental Therapeutics, Vancouver, BC V5Z 1L3, Canada; E-Mail: fcrea@bccrc.ca; 3 Departments of Medical Oncology, BC Cancer Agency, Vancouver, BC V5Z 4E6, Canada; E-Mail: robyn.macfarlane@cdha.nshealth.ca; 4 Departments of Urologic Sciences, Faculty of Medicine, University of British Columbia, Vancouver, BC V6T 1Z3, Canada; 5 Department of Surgery, Faculty of Medicine, University of British Columbia, Vancouver, BC V5Z 4E3, Canada

**Keywords:** microRNA, prostate cancer, metastasis, PSA, castration resistant

## Abstract

MicroRNAs (miRNAs) have emerged as key regulators of numerous biological processes, and increasing evidence suggests that circulating miRNAs may be useful biomarkers of clinical disease. In this study, we sought to identify plasma miRNAs that differentiate patients with metastatic castration resistant prostate cancer (mCRPC) from those with localized prostate cancer (PCa). Pooled plasma samples from patients with localized PCa or mCRPC (25 per group) were assayed using the Exiqon miRNA qPCR panel, and the differential expression of selected candidates was validated using qRT-PCR. We identified 63 miRNAs upregulated in mCRPC *versus* localized PCa, while only four were downregulated. Pearson’s correlation analysis revealed two highly correlated groups: one consisting of miR-141, miR375 and miR-200c and the other including miR151-3p, miR423-3p, miR-126, miR152 and miR-21. A third group, containing miR-16 and miR-205, showed less correlation. One miRNA from each group (miR-141, miR151-3p and miR-16) was used for logistic regression analysis and proved to increase the sensitivity of the prostate-specific antigen (PSA) test alone. While no miRNA alone differentiated localized PCa and mCRPC, combinations had greater sensitivity and specificity. The expression of these 10 candidates was assayed for association with clinical parameters of disease progression through the cBio portal. Our results demonstrate that plasma levels of selected miRNAs are potential biomarkers to differentiate localized PCa and mCRPC.

## 1. Introduction

Prostate cancer (PCa) is the most frequent malignancy amongst males in the Western world3, and it is the second leading cause of cancer-related deaths [1]. Prostate-specific antigen (PSA) testing has increased the proportion of patients diagnosed with localized disease [2]. Treatment options for localized PCa include surgery, radiation and hormonal therapy. For those men who undergo a definitive treatment for localized PCa, a proportion of them will develop recurrent disease, which, after an initial period of hormone responsiveness, will inevitably progress to a castration-resistant state. Metastatic castration-resistant PCa (mCRPC) is still an incurable disease. Given the significant heterogeneity in the behavior of PCa in both the localized and mCRPC states, there is an urgent need to identify biomarkers that will be useful in predicting the progression and outcome of individual tumors.

MicroRNAs (miRNAs) are small RNA molecules consisting of 19–23 nucleotides that play important roles in regulating numerous biological processes [3,4]. It is estimated that miRNAs regulate the expression of at least 60% of all human genes [5], thus accounting for the large number of known human miRNAs [6]. Alterations in miRNA levels can influence cellular processes, such as proliferation, apoptosis and cell cycle regulation, thus providing a link between miRNA dysregulation and cancer [7].

The inherent regulatory role of miRNAs, coupled with the high degree of tissue-specificity of their expression, suggests that they might be informative of disease stage [8,9], making miRNAs attractive candidates as biomarkers. miRNAs are very stable, and they can be readily detected using highly specific and sensitive detection methods (*i.e.*, quantitative RT-PCR) [9,10] in a variety of different sample types. In PCa, miRNAs have been shown to discriminate between healthy individuals and those with cancer [10–14]. In addition, some miRNAs correlate with the risk of disease progression [15] and other predictors of disease outcome, such as the Gleason score and lymph node involvement [12]. Both miRNA-141 and miRNA-375 have been shown to be upregulated in the blood of PCa patients in numerous studies [10,12,14]. Despite the efforts in this field, the degree of overlap amongst the candidate miRNAs identified in these studies is not extensive, perhaps due to the inherent heterogeneity of PCas. As such, there is a need for further investigation to establish a consensus core of blood biomarkers to reliably determine the stage and likelihood of progression of PCa. We thus sought to elucidate the expression levels of miRNAs in the plasma of patients with localized PCa and those with mCRPC as a first step toward identifying novel biomarkers of disease progression.

## 2. Results and Discussion

### 2.1. Detection of Differentially Expressed miRNAs in PCa Patients’ Plasma Samples

The primary goal of this study was to determine if differences in plasma miRNA levels might be useful to differentiate between patients with localized PCa and mCRPC. Of the 742 miRNAs represented on the Exiqon miRNA qPCR panel, 246 had quantification cycle (Cq) values <35 in at least one of the two pooled samples and, thus, were used for further analysis (Supplementary Table S1). Comparisons of mCRPC *versus* localized PCa were carried out to identify miRNAs showing ≥2 Cq differences in expression (Supplementary Table S1). Using this threshold, there were 63 miRNAs upregulated in mCRPC *versus* localized PCa. Surprisingly, considerably fewer miRNAs—only four—were downregulated in mCRPC *versus* localized PCa.

### 2.2. Deregulated Expression of Plasma miRNAs in Prostate Tumor Tissue

The miRNAs detected in plasma may arise from a number of different sources, such as circulating blood cells, circulating tumor cells or exosomes released from the tumor. We thus determined if the differentially expressed plasma miRNAs showed similarly deregulated expression in a previously analyzed paired xenograft model of human non-metastatic and metastatic PCa [16]. For this comparison, we used a threshold of ≥1 Cq increased expression, which expanded the list of differentially expressed plasma miRNAs to include 123 candidates (Supplementary Table S1). Comparison of these two groups revealed 15 commonly increased miRNAs in metastatic PCa (Table 1). We next carried out a similar comparison using miRNAs that were decreased ≥1 Cq in mCRPC *versus* localized PCa (Supplementary Table S1). Of these, four were downregulated in both the mCRPC plasma samples analyzed in this study and the metastatic tumor tissue xenografts (Table 1).

### 2.3. Expression Analysis of Selected miRNAs in Individual Plasma Samples

As the primary goal of this study was to identify miRNAs differentiating localized PCa from mCRPC, we selected a number of candidates for validation of expression differences in the 50 individual plasma samples based on the analyses described above and our pooled plasma miRNA expression profiles. Included in this list were miRNAs-141, -152 [17] and -375, which have previously been shown to be potential biomarkers [12,14], as well as miR-16, -21, -126, -151-3p, -200c, -205 and -423-3p (selected from Supplementary Table S1). A dilution series of the reference RNA was used to ensure that the amplification efficiency of the qPCR primer set for each miRNA had Cq ≤35 in more than 80% of the samples.

**Figure 1:**
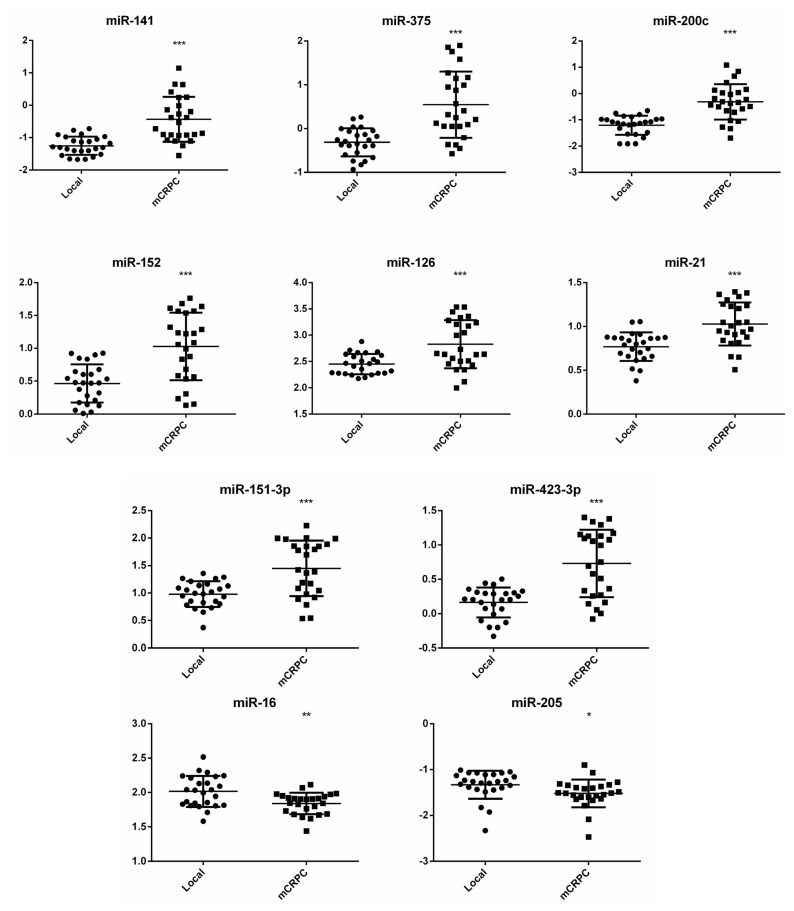
Quantitative RT-PCR analysis of upregulated and downregulated miRNAs in individual plasma samples from patients with localized (local) prostate cancer (PCa) or metastatic castration-resistant PCa (mCRPC). Quantification cycle (Cq) values were converted to log base amounts based on the cell line reference expression as outlined in the Materials section. Statistical analysis was carried out using the Student’s *t*-test. * indicates *p* < 0.05, ** indicates *p* < 0.01 and *** indicates *p* < 0.001.

**Figure 2:**
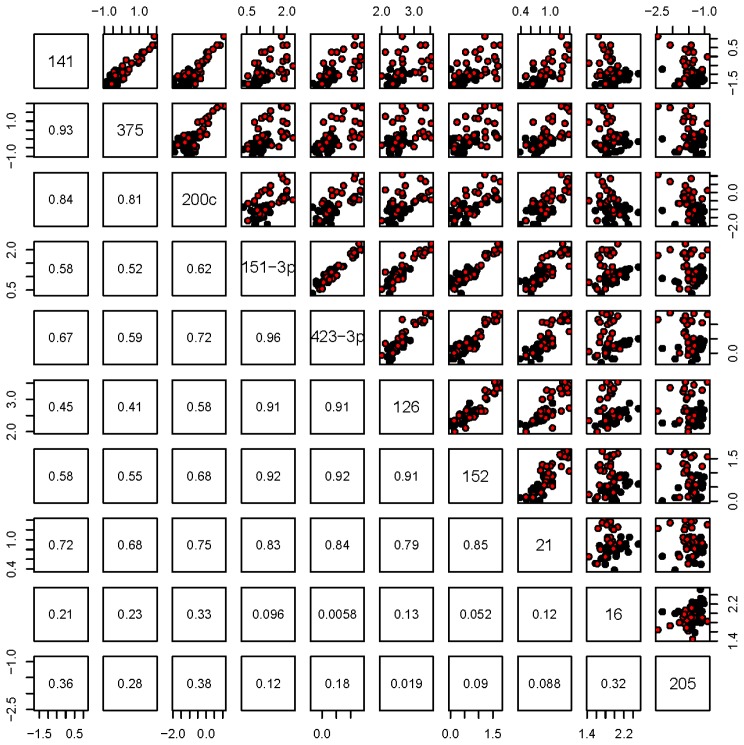
Pearson’s correlation amongst the validated differentially expressed miRNAs. Black dots represent localized PCa, and red dots represent mCRPC.

**Figure 3:**
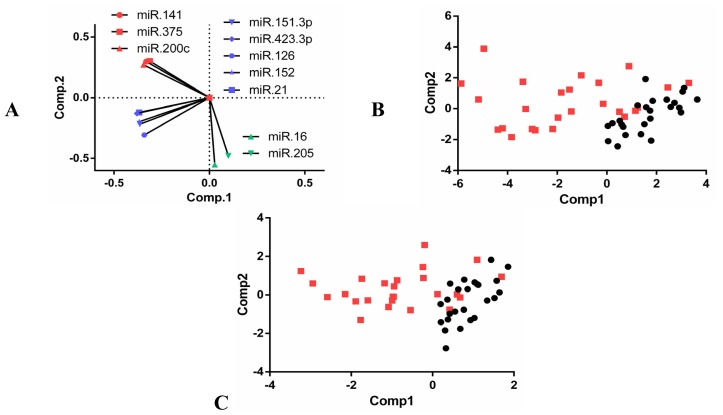
Principle Component Analysis (PCA) on the ten validated differentially expressed miRNAs. (**A**) Visual representation of the highly correlated pattern observed in the Pearson’s correlation; (**B**) PCA using all 10 miRNAs is able to distinguish localized PCa from mCRPC; although (**C**) PCA using a combination of only miR-141 + miR-151-3p + miR-16 shows a similar ability to separate the two conditions.

Figure 1 shows the normalized miRNA levels for the selected up- and down-regulated candidates in the individual plasma samples isolated from patients with localized PCa or mCRPC. In confirmation of the data from the panel assay, the expression level of each selected miRNA in the mCRPC group was significantly different in the localized PCa patients. Among the samples, miR-141, -375 and -200c showed similar patterns of expression, and analyses of the Pearson’s correlation amongst pairs of these three candidates were the highest (Figure 2). A second group showing high correlation included miR-126, -21, -151-3p, -152 and -423-3p (Figure 2). The two downregulated miRNAs included in this analysis, miR-16 and miR-205, showed no correlation (Figure 2). Principle Component Analysis (PCA; Figure 3) depicts the three groups of miRNAs and demonstrates that two components are able to distinguish localized PCa from mCRPC, with each miRNA contributing in the manner predicted by the correlations. Importantly, combining one miRNA from each group was comparable to all ten in differentiating between localized PCa and mCRPC.

**Figure 4:**
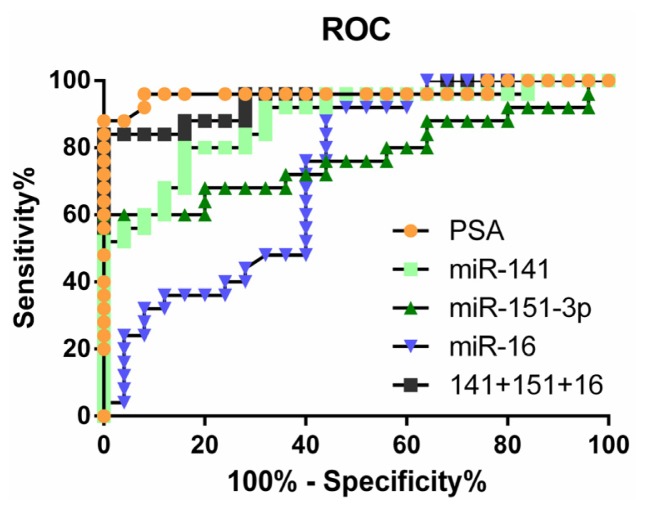
Receiver operating characteristic (ROC) curves to assess the utility of selected individual miRNAs compared with the combination or with PSA to differentiate localized PCa from mCRPC. ROC curves for miR-141 (light squares), miR-151-3p (triangle), miR-16 (upside-down triangle), the combination of all three miRs (dark square) and PSA (circle).

Based on these results, we determined the minimal number and combination of miRNAs required to differentiate localized PCa from mCRPC with high specificity and sensitivity. To do this, we selected one miRNA from each of the three groups shown in Figure 3A and used logistic regression to calculate the probability that one miRNA or a combination of three miRNAs could distinguish mCRPC from localized PCa. The error values were determined using leave-one-out cross-validation (LOOCV); Akaike’s Information Criterion (AIC) values were calculated and probabilities were plotted to generate receiver operating characteristic (ROC) curves for each miRNA alone, as well as the combination (Figure 4). The area under the curve (AUC) was calculated for each (Table 2). Due to the high correlation of expression, the combination of miRNAs in a group (e.g., miR-141 + miR-375) was less useful for differentiating mCRPC from localized PCa than a single miRNA (data not shown). We assessed different combinations of three miRNAs, each from one group. Surprisingly, the combination of the best single discriminators from each group did not yield the highest AUC value (data not shown). To the contrary, miR-141, miR-151-3p and miR-16 in combination yielded an AUC of 0.944, slightly below that for PSA (0.964). Interestingly, combining the PSA test and mir-141-1513p-16 test increased PSA sensitivity by 8%.

### 2.4. Clinical Significance of Differentially Expressed miRNAs

Finally, we determined if any of the ten validated differentially expressed plasma miRNAs correlated with clinicopathological parameters of PCa in a recently analyzed large cohort of PCa patients (Table 3) [18]. Our results indicate that and miR-205 (downregulated in mCRPC) is associated with a lower Gleason Score and a lower probability of both biochemical recurrence and clinically evident metastatic events after prostatectomy. To the contrary, miR-141, 151-3p, 152 and 423-3p are associated with a poorer outcome and/or a higher Gleason Score. MiR-141 and mir-152 seemed to discriminate a rare, but highly significant sub-group of PCa patients, *i.e.*, individuals with a higher than 90% probability of recurrence after prostatectomy. MiR-423-3p and miR-205 emerged as novel and particularly meaningful prognostic factors, since they are correlated with several clinical parameters.

### 2.5. Discussion

We previously demonstrated, using human metastatic *versus* non-metastatic PCa xenograft tumor tissue lines, a significant number of differentially expressed miRNAs associated with PCa metastasis [16]. MiRNAs originating from tumor tissue can enter the circulation and function as reliable biomarkers in human cancers [10]. Compared with proteins or mRNAs, it has been shown that miRNAs can stably exist in plasma samples even after 24 h of incubation at room temperature [10]. We therefore initiated this study to compare levels of circulating miRNAs in patients with treatment-naive localized PCa or mCRPC.

The vast majority of differentially expressed miRNAs were found at higher levels, rather than lower levels, in mCRPC *versus* localized PCa. The reason for this is not immediately clear. One possibility is that many of these might be tumor-derived and, thus, reflect the increased tumor burden and potentially a larger number of circulating cancer cells. Among the upregulated miRNAs selected for more in-depth analysis, miR-141 and miR-375 were the first ones reported as a circulating miRNAs in PCa [10,19]. Upregulation of miR-141 and miR-375 has been reported in PCa *versus* benign tissues [19]. In this study, we also observed increased expression of miR-141 and miR-375 in plasma from patients with mCRPC compared to those with localized PCa.

Expression of miR-126, -151-3p, -152, -423-3p and -21 was also upregulated in mCRPC *versus* localized PCa, but the pattern of upregulation was different from that of miR-141/375, as shown by the lower Pearson’s correlation (Figure 2). miR-21 is an androgen receptor (AR)-regulated miRNA that promotes PCa growth [20]. In addition, miR-21 levels are increased in the serum of docetaxel-resistant patients [13]. Integrating PSA levels with miRNA-21 and miR-141 profiles significantly increases the positive predictive value for PCa compared to that of PSA alone [21]. We observed that both miR-126 and miR-152, upregulated in this study, were also upregulated in metastatic *versus* non-metastatic PCa tumor tissue xenografts [16]. Interestingly, in conflict with our previous and current findings, miR-126 has been reported to be a tumor suppressor [22], and miR-152 was shown to be decreased in PCa [23]. Given the heterogeneous nature of PCas, the observation that levels of tumor suppressive miRNAs are elevated in the plasma raises the interesting possibility that cells expressing elevated levels of these miRNAs are actively shed from the tumor. In contrast to the upregulated plasma miRNAs in the mCRPC group, the downregulated miRNAs comprised a much smaller group. In addition, the majority of the downregulated plasma miRNAs are downregulated in PCa tissues [16].

Amongst the validated miRNAs upregulated in mCRPC, we observed variations in the expression patterns suggesting that those with highly correlated expression may be under similar transcriptional control or be secreted into the circulation by the same mechanisms. These variations in expression patterns divide mCRPC into subgroups such that expression of certain miRNAs may be linked to metastasis, some to the status of AR signaling (*i.e.*, miR-141 and miR-375) and some to both. ROC curve analysis of miRNA expression and regression analysis of the AUC indicated that no single over-expressed plasma miRNA was useful for differentiating between localized PCa and mCRPC. In contrast, certain combinations of differentially expressed miRNAs (*i.e.*, miR-141 + miR151-3p + miR-16) were almost as good as PSA levels in discriminating between mCRPC and localized PCa, and combining the miRNA test to the classical PSA dosage might result in a relevant sensitivity gain. Those results may pave the way to larger clinical studies, assessing the feasibility of coupling miRNA dosage with the PSA test in the clinical setting.

Finally, we sought to determine if the changes in plasma miRNA levels associated with disease progression had any relation to clinical parameters in a large patient cohort [18]. Our findings revealed that altered levels of three miRNAs not previously identified as blood biomarkers (miR-151-3p, miR-423-3p and miR-205) correlated with several clinical parameters.

## 3. Experimental Section

### 3.1. Human Plasma Samples

After obtaining institutional REB approval and informed consent from all study participants, blood samples were drawn at the British Columbia Cancer Agency (BCCA) in Vancouver in accordance with the National Institute of Cancer standard operating procedures for serum and plasma processing [24]. Two cohorts were evaluated: 25 PCa patients with treatment-naive localized disease (localized PCa) and 25 patients with metastatic castration resistant PCa and a rising PSA (mCRPC patients). The demographics of the mCRPC group have been published previously [14], but the characteristics of patients in both the localized cancer and mCRPC groups are provided in Table 4. All blood samples were collected in ethylenediaminetetraacetic acid (EDTA)-containing tubes and refrigerated. Samples were processed within 4 h of collection, and the resultant plasma samples were stored in a −80 °C freezer until RNA extraction.

### 3.2. RNA Extraction, cDNA Preparation, qRT-PCR

RNA was extracted from 200 μL of plasma using the miRNeasy mini kit (Qiagen, Toronto, Canada), following the manufacturer’s instructions with the following modifications: Trizol LS (600 μL) (Invitrogen, Burlington, Canada) was used for RNA extraction instead of Qiazol, glycogen was added as an RNA carrier, an extra chloroform extraction step was added following the phenol/chloroform extraction and an additional wash step with RPE buffer was added. The RNA was eluted in 50 μL of nuclease-free water, and 5 μL of each sample was pooled to yield two groups of samples (localized PCa and mCRPC) for assay using the human microRNA qPCR panel (Exiqon, Woburn, MA, USA).

Eight microliters of each pooled RNA sample was reverse transcribed using the Universal cDNA Synthesis Kit (Exiqon, Woburn, MA, USA), according to the manufacturer’s instructions. The resultant cDNA was diluted and mixed with the microRNA LNA PCR primers and SYBR Green master mix (Exiqon, Woburn, MA, USA). Quantitative PCR (qPCR) was done on the ABIPrism 7900HT (Applied Biosystems, Streetsville, Canada), following the manufacturer’s instructions.

RNA isolated from the prostate cell lines, BPH1 and 22Rv1, was used as a reference RNA sample to generate a standard curve for expression of each miRNA, as well as to calculate their relative expression. The quantity of each miRNA was calculated, and logarithmic values were plotted. For normalization of the miRNA qPCR data, we screened the expression of all miRNAs represented in the panel and selected the miRNA with the minimal variation between the 2 groups (mir-30e). Mir-30e stability was confirmed by qPCR and compared to the levels of mir-16, which is frequently used as a reference miRNA. Based on this analysis, we selected mir-30e as an internal control, since mir-30e expression was very stable with no significant differences between the groups in a Student’s *t*-test (Figure S1).

### 3.3. Analysis of Differentially Expressed miRNAs in Human Tumor Tissues

We determined whether the miRNAs differentially expressed in plasma also had altered expression levels in PCa tissues analyzed in a previously published dataset [18]. The raw data was obtained from GEO (accession number GSE21036) and converted to a Z-score. A Z-score ratio of greater than 1.96 between primary and metastatic PCa groups was considered significantly different. We also compared the differentially expressed miRNAs identified with those differentially expressed in a metastatic PCa xenograft [16].

### 3.4. Statistical Analysis

The correlation between pairs of miRNA expression levels was evaluated using Pearson’s correlation [26]. Logistic regression analysis was done using the glm package of R [27] and calculated leave-one-out cross-validation (LOOCV) using cv.glm for selecting the best combination. Principle component analysis (PCA) was also carried out using R. All other statistical analyses (Mann-Whitney U test, student’s *t*-test, ROC curve and AUC) were carried out using GraphPad Prism.

## 4. Conclusions

In this study, we compared plasma levels of miRNAs in localized PCa *versus* mCRPC and identified selected combinations of miRNAs that appear to be suitable for differentiating localized PCa and mCRPC. Larger patient cohorts are required to confirm their potential utility as biomarkers in PCa detection and stratification. Monitoring expression levels of plasma miRNAs may also be useful as predictive and prognostic biomarkers, particularly those associated with AR signalling, like miR-21, given the recent availability of treatments for mCRPC, like abiraterone acetate [28] and enzalutamide (MDV3100) [29], which target persistent AR signalling more effectively.

## Supplementary Information



## Figures and Tables

**Table 1:** Cq values for miRNAs that show similar trends in expression differences in plasma from patients with localized PCa *versus* mCRPC and in non-metastatic *versus* metastatic tumor tissue.

Increased miRNA	Localized	mCRPC	Notes 1
miRNA-125b-2 *	ND	33.88	>2
miRNA-136	34.58	31.35	>2
miRNA-151-3p	31.61	29.26	>2
miRNA-200a	34.27	31.52	>2
miRNA-744a *	38.09	34.51	>2
miRNA-9	39.09	34.79	>2
miRNA-9 *	ND	34.12	>2
miRNA-99a	34.28	32.02	>2
Let-7d	33.21	31.37	>1
miRNA-126	26.49	24.93	>1
miRNA-142-5p	30.35	29.16	>1
miRNA-18b	30.14	28.98	>1
miRNA-27a	29.07	27.31	>1
miRNA-27b	29.15	27.25	>1
miRNA-30a *	35.48	34.33	>1
Decreased miRNA	Local	mCRPC	Notes
miRNA-205	32.43	34.90	>2
miRNA-106b	30.38	31.50	>1
miRNA-16	22.64	23.97	>1
miRNA-363	29.93	31.08	>1

1 >2 or >1 refer to Cq differences in expression in the mCRPC *versus* localized PCa group (Supplemental Table S1). miRNAs showed increased or decreased expression in a metastatic prostate tumor xenograft model, as described previously [16].

**Table 2:** ROC analysis to assess the specificity and sensitivity of individual or pairs of miRNAs to differentiate between localized PCa and mCRPC. Sensitivity and specificity refer to the threshold value with the highest likelihood ratio.

	AUC	95% Confidence interval	Sensitivity	Specificity
miR-141	0.8784	0.7832 to 0.9736	56%	96%
miR-375	0.8576	0.7499 to 0.9653	56%	96%
miR-151-3p	0.7696	0.6310 to 0.9082	60%	96%
miR-126	0.7488	0.6100 to 0.8876	48%	96%
miR-16	0.7160	0.5708 to 0.8612	24%	96%
miR-205	0.7536	0.6094 to 0.8978	60%	88%
miR141 + 151-3p + 16	0.9440	0.8795 to 1.008	84%	96%
PSA	0.9640	0.9040 to 1.024	88%	96%
PSA + miR141 + 151-3p + 16	0.9680	0.9087 to 1.027	96%	96%

**Table 3:** Association of miRNAs with clinical parameters of PCa progression. We analyzed the correlation between clinical parameters and expression levels of 11 miRNAs (3 downregulated and 8 upregulated in mCRCPC *versus* localized PCa plasma samples). Selected miRNAs were the ones not previously correlated with PCa progression. *p* values are considered significant when *p* < 0.05/11 (Bonferroni correction for multiple comparisons). For each parameter, we show the *p* value (NS, non-significant) and the absolute value of gene expression in the 2 sub-categories (e.g., low *versus* high Gleason score). For each sub-category, the number of patients analyzed is shown in brackets. Significant results are presented in bold font. Biochemical recurrence is defined as PSA ≥ 0.2 ng/mL on two occasions after prostatectomy.

		Upregulated in CRPC				Downregulated in CRPC
	
		miR-141	miR-151-3p	miR-152	miR-423-3p	miR-205
Pathological Gleason score	*p*-value	NS	NS	NS	**0.002**	**0.004**
	7 or less (87)	13.186	7.734	7.165	**3.709**	**10.68**
	8 or more (19)	13.389	8.152	6.919	**4.26**	**7.093**

Lymph node involvement	*p*-value	NS	NS	NS	**0.003**	NS
	Abnormal N1 (13)	13.692	8.193	6.707	**4.423**	5.744
	Normal N0 (79)	13.139	7.762	7.164	**3.757**	10.339

Time to tumor recurrence	*p*-value	**0.003**	NS	**0.01**	NS	NS
	<1 year (19)	**13.641**	8.101	**6.736**	4.192	7.218
	>1 year (81)	**13.144**	7.754	**7.181**	3.727	10.568

PSA at diagnosis	*p*-value	NS	NS	NS	**0.003**	0.005
	<20 (100)	13.198	7.787	7.125	**3.79**	10.17
	>20 (11)	13.603	8.151	6.568	**4.337**	5.748

**Probability of freedom of biochemical recurrence (5 years)**	*p*-value	**<0.001**	NS	**0.002**	NS	NS
	<90% (21)	**13.715**	8.118	**6.647**	4.133	6.77
	>90% (74)	**13.155**	7.713	**7.203**	3.678	10.74

**Metastatic events**	*p*-value	NS	NS	NS	**<0.001**	**<0.001**
	Clinical (19)	13.149	8.145	6.466	**4.529**	**5.045**
	none (94)	13.231	7.764	7.169	**3.723**	**10.657**

**Biochemical recurrence event**	*p*-value	NS	**<0.001**	NS	**<0.001**	**0.006**
	yes (27)	13.36	**8.125**	6.923	**4.177**	**8.03**
	no (80)	13.189	**7.711**	7.162	**3.685**	**10.629**

**Table 4:** Baseline and clinical demographics of the patients with localized PCa and mCRPC from whom plasma samples were obtained.

**Clinically Localized Prostate Cancer Patients (*N*= 25)**	
Median Age = 67 years; Range = 51–82 years	

Patient Characteristics	Number of Patients
T Stage	
T1C	11
T2a	7
T2b	6
T3	1

PSA at Diagnosis	
≤10	15
>10 to ≤20	9
>20	1

Gleason Score	
≤6	7
7	15
≥8	3

Risk Group [25]	
Low	4
Intermediate	17
High	4

**mCRPC Patients (*N*= 25)**	
Median Age = 73 years; Range = 45–86 years	

Patient Characteristics	Number of Patients
PSA	
<50	6
>50 to ≤300	14
≥300	5

Metastatic Site	
Bone	17
Lymph Node ± Other	5
Other	3

Therapy at Time of Sampling	
Observation *	15 (1 was not on LHRH-A)
Complete Androgen Blockade	4
Prednisone ± LHRH-A	4
Abiraterone	2

* For patients on observation, the assumption is that they were either on LHRH-A or had had previous orchiectomy.

## References

[1] Jemal A., Siegel R., Ward E., Hao Y., Xu J., Murray T., Thun M.J. (2008). Cancer statistics, 2008. CA Cancer J. Clin.

[2] Kohli M., Tindall D.J. (2010). New developments in the medical management of prostate cancer. Mayo Clin. Proc.

[3] Bartel D.P. (2004). MicroRNAs: Genomics, biogenesis, mechanism, and function. Cell.

[4] Bushati N., Cohen S.M. (2007). microRNA functions. Annu. Rev. Cell Dev. Biol.

[5] Friedman R.C., Farh K.K., Burge C.B., Bartel D.P. (2009). Most mammalian mRNAs are conserved targets of microRNAs. Genome Res.

[6] Kozomara A., Griffiths-Jones S. (2011). miRBase: Integrating microRNA annotation and deep-sequencing data. Nucleic Acids Res.

[7] Miska E.A. (2005). How microRNAs control cell division, differentiation and death. Curr. Opin. Genet. Dev.

[8] Lu J., Getz G., Miska E.A., Alvarez-Saavedra E., Lamb J., Peck D., Sweet-Cordero A., Ebert B.L., Mak R.H., Ferrando A.A. (2005). MicroRNA expression profiles classify human cancers. Nature.

[9] Tam W. (2008). The emergent role of microRNAs in molecular diagnostics of cancer. J. Mol. Diagn.

[10] Mitchell P.S., Parkin R.K., Kroh E.M., Fritz B.R., Wyman S.K., Pogosova-Agadjanyan E.L., Peterson A., Noteboom J., O’Briant K.C., Allen A. (2008). Circulating microRNAs as stable blood-based markers for cancer detection. Proc. Natl. Acad. Sci. USA.

[11] Lodes M.J., Caraballo M., Suciu D., Munro S., Kumar A., Anderson B. (2009). Detection of cancer with serum miRNAs on an oligonucleotide microarray. PLoS One.

[12] Brase J.C., Johannes M., Schlomm T., Falth M., Haese A., Steuber T., Beissbarth T., Kuner R., Sultmann H. (2011). Circulating miRNAs are correlated with tumor progression in prostate cancer. Int. J. Cancer.

[13] Zhang H.L., Yang L.F., Zhu Y., Yao X.D., Zhang S.L., Dai B., Zhu Y.P., Shen Y.J., Shi G.H., Ye D.W. (2011). Serum miRNA-21: Elevated levels in patients with metastatic hormone-refractory prostate cancer and potential predictive factor for the efficacy of docetaxel-based chemotherapy. Prostate.

[14] Selth L.A., Townley S., Gillis J.L., Ochnik A.M., Murti K., Macfarlane R.J., Chi K.N., Marshall V.R., Tilley W.D., Butler L.M. (2012). Discovery of circulating microRNAs associated with human prostate cancer using a mouse model of disease. Int. J. Cancer.

[15] Moltzahn F., Olshen A.B., Baehner L., Peek A., Fong L., Stoppler H., Simko J., Hilton J.F., Carroll P., Blelloch R. (2011). Microfluidic-based multiplex qRT-PCR identifies diagnostic and prognostic microRNA signatures in the sera of prostate cancer patients. Cancer Res.

[16] Watahiki A., Wang Y., Morris J., Dennis K., O’Dwyer H.M., Gleave M., Gout P.W. (2011). MicroRNAs associated with metastatic prostate cancer. PLoS One.

[17] Zhu C., Li J., Ding Q., Cheng G., Zhou H., Tao L., Cai H., Li P., Cao Q., Ju X. (2013). miR-152 controls migration and invasive potential by targeting TGFalpha in prostate cancer cell lines. Prostate.

[18] Taylor B.S., Schultz N., Hieronymus H., Gopalan A., Xiao Y., Carver B.S., Arora V.K., Kaushik P., Cerami E., Reva B. (2010). Integrative genomic profiling of human prostate cancer. Cancer Cell.

[19] Brase J.C., Wuttig D., Kuner R., Sultmann H. (2010). Serum microRNAs as non-invasive biomarkers for cancer. Mol. Cancer.

[20] Ribas J., Ni X., Haffner M., Wentzel E.A., Salmasi A.H., Chowdhury W.H., Kudrolli T.A., Yegnasubramanian S., Luo J., Rodriguez R. (2009). miR-21: An androgen receptor-regulated microRNA that promotes hormone-dependent and hormone-independent prostate cancer growth. Cancer Res.

[21] Hao Y., Zhao Y., Zhao X., He C., Pang X., Wu T.C., Califano J.A., Gu X. (2011). Improvement of prostate cancer detection by integrating the PSA test with miRNA expression profiling. Cancer Invest.

[22] Saito Y., Friedman J.M., Chihara Y., Egger G., Chuang J.C., Liang G. (2009). Epigenetic therapy upregulates the tumor suppressor microRNA-126 and its host gene EGFL7 in human cancer cells. Biochem. Biophys. Res. Commun.

[23] Szczyrba J., Loprich E., Wach S., Jung V., Unteregger G., Barth S., Grobholz R., Wieland W., Stohr R., Hartmann A. (2010). The microRNA profile of prostate carcinoma obtained by deep sequencing. Mol. Cancer Res.

[24] U. S. National Institutes of Health http://edrn.nci.nih.gov/resources/standardoperating-procedures/biological-specimens.

[25] D’Amico A.V., Whittington R., Malkowicz S.B., Schultz D., Blank K., Broderick G.A., Tomaszewski J.E., Renshaw A.A., Kaplan I., Beard C.J. (1998). Biochemical outcome after radical prostatectomy, external beam radiation therapy, or interstitial radiation therapy for clinically localized prostate cancer. JAMA.

[26] (2012). Graph Pad Prism Software.

[27] Institute for Statistics and Mathematics of WU The R Project for Statistical Computing.

[28] De Bono J.S., Logothetis C.J., Molina A., Fizazi K., North S., Chu L., Chi K.N., Jones R.J., Goodman O.B., Saad F. (2011). Abiraterone and increased survival in metastatic prostate cancer. N. Engl. J. Med.

[29] De Bono J.S., Fizazi K., Saad F., Taplin M.-E., Sternberg C.N., Miller K., Mulders P., Chi K.N., Armstrong A.J., Hirmand M. (2012). Primary, secondary, and quality-of-life endpoint results from the phase III AFFIRM study of MDV3100, an androgen receptor signaling inhibitor. J. Clin. Oncol..

