# Supplementary material for: Plasma miRNAs as Biomarkers to Identify Patients with Castration-Resistant Metastatic Prostate Cancer
Source: Int J Mol Sci. 2013 Apr 10;14(4):7757–70. doi: 10.3390/ijms14047757 (PMC3645714; doi:10.3390/ijms14047757)
Supplement: Supplementary file 1 [file ijms-14-07757-s001.pdf]

Supplementary Information

Figure S1. Comparison between mir-30e and mir-16 stability in plasma samples from the 2 analyzed groups. *T* test *p* value = 0.1436 (mir-30e) *vs*. 0.002 (mir-16). Differences between means: 0.119 ± 0.080 (mir-30e) *vs*. (−0.175) ± 0.055 (mir-16). ** $p < 0.01$ (*T* test).

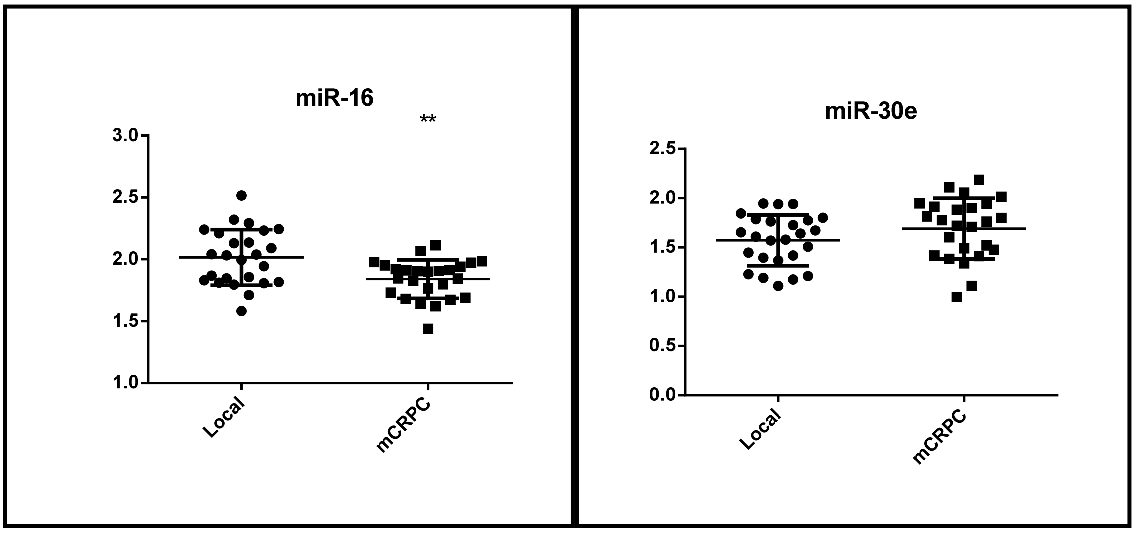

© 2013 by the authors; licensee MDPI, Basel, Switzerland. This article is an open access article distributed under the terms and conditions of the Creative Commons Attribution license (http://creativecommons.org/licenses/by/3.0/).
